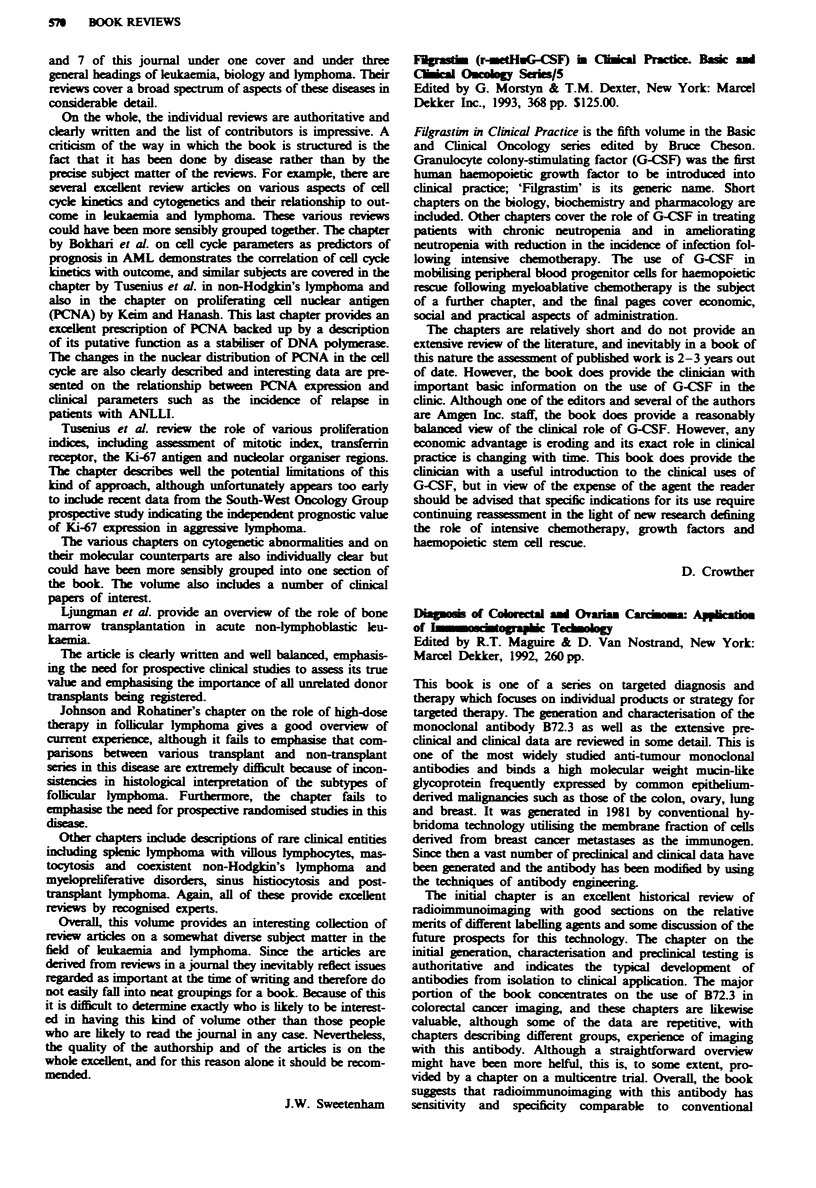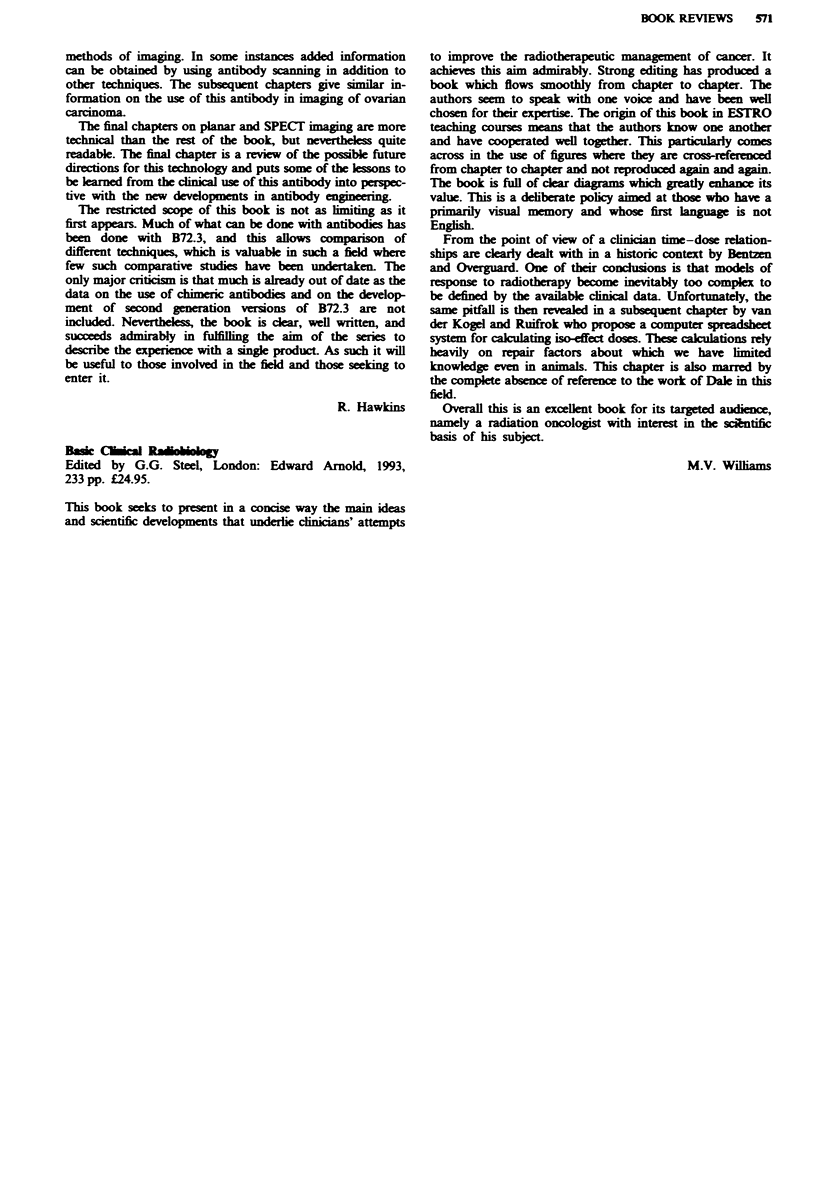# Diagnosis of colorectal and ovarian carcinoma: application of immunoscintographic technology

**Published:** 1994-09

**Authors:** R. Hawkins


**Diagnosis of Colorectal and Ovarian Carcinoma: Application of Immunoscintographic Technology**

Edited by R.T. Maguire & D. Van Nostrand, New York: Marcel Dekker, 1992, 260 pp.

This book is one of a series on targeted diagnosis and therapy which focuses on individual products or strategy for targeted therapy. The generation and characterisation of the monoclonal antibody B72.3 as well as the extensive preclinical and clinical data are reviewed in some detail. This is one of the most widely studied anti-tumour monoclonal antibodies and binds a high molecular weight mucin-like glycoprotein frequently expressed by common epithelium-derived malignancies such as those of the colon, ovary, lung and breast. It was generated in 1981 by conventional hybridoma technology utilising the membrane fraction of cells derived from breast cancer metastases as the immunogen. Since then a vast number of preclinical and clinical data have been generated and the antibody has been modified by using the techniques of antibody engineering.

The initial chapter is an excellent historical review of radioimmunoimaging with good sections on the relative merits of different labelling agents and some discussion of the future prospects for this technology. The chapter on the initial generation, characterisation and preclinical testing is authoritative and indicates the typical development of antibodies from isolation to clinical application. The major portion of the book concentrates on the use of B72.3 in colorectal cancer imaging, and these chapters are likewise valuable, although some of the data are repetitive, with chapters describing different groups, experience of imaging with this antibody. Although a straightforward overview might have been more helful, this is, to some extent, provided by a chapter on a multicentre trial. Overall, the book suggests that radioimmunoimaging with this antibody has sensitivity and specificity comparable to conventional methods of imaging. In some instances added information can be obtained by using antibody scanning in addition to other techniques. The subsequent chapters give similar information on the use of this antibody in imaging of ovarian carcinoma.

The final chapters on planar and SPECT imaging are more technical than the rest of the book, but nevertheless quite readable. The final chapter is a review of the possible future directions for this technology and puts some of the lessons to be learned from the clinical use of this antibody into perspective with the new developments in antibody engineering.

The restricted scope of this book is not as limiting as it first appears. Much of what can be done with antibodies has been done with B72.3, and this allows comparison of different techniques, which is valuable in such a field where few such comparative studies have been undertaken. The only major criticism is that much is already out of date as the data on the use of chimeric antibodies and on the development of second generation versions of B72.3 are not included. Nevertheless, the book is clear, well written, and succeeds admirably in fulfilling the aim of the series to describe the experience with a single product. As such it will be useful to those involved in the field and those seeking to enter it.

R. Hawkins